# Microbial Production of Isoprenoids Enabled by Synthetic Biology

**DOI:** 10.3389/fmicb.2013.00075

**Published:** 2013-04-04

**Authors:** Cheryl M. Immethun, Allison G. Hoynes-O’Connor, Andrea Balassy, Tae Seok Moon

**Affiliations:** ^1^Department of Energy, Environmental & Chemical Engineering, Washington University in St. LouisSaint Louis, MO, USA

**Keywords:** microbial biosynthesis, synthetic biology, metabolic engineering, isoprenoids, health industry

## Abstract

Microorganisms transform inexpensive carbon sources into highly functionalized compounds without toxic by-product generation or significant energy consumption. By redesigning the natural biosynthetic pathways in an industrially suited host, microbial cell factories can produce complex compounds for a variety of industries. Isoprenoids include many medically important compounds such as antioxidants and anticancer and antimalarial drugs, all of which have been produced microbially. While a biosynthetic pathway could be simply transferred to the production host, the titers would become economically feasible when it is rationally designed, built, and optimized through synthetic biology tools. These tools have been implemented by a number of research groups, with new tools pledging further improvements in yields and expansion to new medically relevant compounds. This review focuses on the microbial production of isoprenoids for the health industry and the advancements though synthetic biology.

## Introduction

Microbial biosynthesis of natural products provides advantages over biomass extraction and chemical synthesis. The native hosts of the natural products, usually plants, grow slowly with differences in the plant’s climate and geography, leading to detrimental variations in the product concentration and composition (Chang and Keasling, 2006; Asadollahi et al., 2008; Engels et al., 2008). In addition, biomass extraction often requires substantial energy and resource consumption for miniscule product recovery (Martin et al., 2003; Shiba et al., 2007; Ajikumar et al., 2008). Chemical synthesis struggles to create the natural product’s integral complexity (Chang and Keasling, 2006; Engels et al., 2008; Nakagawa et al., 2011). It also requires significant amounts of energy while producing toxic by-products (Chemler et al., 2006; Winter and Tang, 2012; Wu et al., 2013). Microbial biosynthesis of natural products can help avert these problems. The biosynthetic pathway from the native host is redesigned in a tractable platform organism, often *Escherichia coli* or *Saccharomyces cerevisiae*, which serves as a microbial cell factory (Shiba et al., 2007; Ajikumar et al., 2008; Albertsen et al., 2011; Du et al., 2011; Misawa, 2011). The microbes can utilize inexpensive carbon sources with short doubling times to produce highly functionalized and value-added products with no toxic by-products (Chemler et al., 2006; Ajikumar et al., 2008; Tang and Zhao, 2009; Hong and Nielsen, 2012). Microbial biosynthesis is well suited for the production of many natural products, including isoprenoids.

Isoprenoids are important secondary metabolites for the health industry. Built from five carbon isoprene units that are cyclized, rearranged, and adorned in a multitude of ways, isoprenoids, sometimes called terpenoids, include more than 40,000 structurally unique compounds (Chang and Keasling, 2006; Ajikumar et al., 2008; Farhi et al., 2011). Terpenoids are classified based on their number of isoprene units. Monoterpenes consist of two isoprene units, with sesquiterpenes, diterpenes, triterpenes, and carotenoids (or tetraterpenes) built from three, four, six, and eight isoprene units, respectively (Withers and Keasling, 2007; Asadollahi et al., 2008; Misawa, 2011; Walter and Strack, 2011). Important compounds for the health industry can be found in many of the terpenoids classes, including the carotenoid lycopene, the sesquiterpene artemisinin, the diterpene paclitaxel, and triterpene herbal medicines (Das et al., 2007; Ajikumar et al., 2008; Asadollahi et al., 2008; Misawa, 2011).

Synthetic biology tools can help boost the delivery of isoprenoids to market. While the natural biosynthetic pathway could be simply transferred to an industrially suited host, such as *E. coli* or *S. cerevisiae*, the final titers of the desired product would become economically feasible when the pathway is rationally designed, built, and optimized (Klein-Marcuschamer et al., 2007; Maury et al., 2008; Anthony et al., 2009). While reasonable titers for the commercial-scale production are currently unknown, a review indicates that 0.5 g/l is an adequate starting point for high value compounds (Ajikumar et al., 2008). Innovations in genomics and systems biology have facilitated the ability to engineer biology for commercial applications through a set of clear steps (Ajikumar et al., 2008; Keasling, 2012). First, the metabolic pathways needed to produce the desired products are selected. Second, a host suitable for industrial production and genetic manipulation is chosen. Third, what must be redesigned for the pathway and host to operate together is determined, followed by optimization so that production can become commercially relevant (Jarboe et al., 2010). These steps have been implemented by a number of research groups for the creation of biofuels, commodity chemicals, and products for the health industry. This review focuses on the microbial production of isoprenoids for the health industry and the advancements through synthetic biology. Four of the 10 isoprenoids discussed (Table 1) are currently manufactured or will be manufactured in the near future.

**Table 1:** **Isoprenoid production**.

Isoprenoid	Approach^1^	Microbial production (fold improvement)	Microbe	Natural source and extraction	Chemical synthesis
Amorphadiene^2^	Express heterologous pathway in two operons and codon-optimize amorphadiene synthase	24 mg caryophyllene equivalent/l (300-fold) (Martin et al., 2003)	*E. coli*	*Artemesia annua* 0.01–1.0% of dry leaf weight (Liu et al., 2006)	29–42% Overall yield (Zhu and Cook, 2012)
	Redesign the mevalonate pathway to increase FPP and express *Artemisia annua*’s amorphadiene synthase and cytochrome P450	153 mg/l (500-fold) (Ro et al., 2006)	*S. cerevisiae*	
	Identify the limiting reaction enzymes and balance gene expression through plasmid copy number and promoter strength	293 mg/l (7-fold) (Anthony et al., 2009)	*E. coli*	
	Overexpress every enzyme in the mevalonate pathway as well as modify fermentation conditions	40 g/l (250-fold) (Westfall et al., 2012)	*S. cerevisiae*	
	Express heterologous pathway in a strain of *Streptomyces avermitilis* with minimized genome	30 mg/l (from 0 mg/l) (Komatsu et al., 2010)	*S. avermitilis*	
	Truncate and deregulate HMG1 and co-localize heterologous FDP synthase and amorphadiene synthase to the mitochondria	20 mg/l (20-fold) (Farhi et al., 2011)	*S. cerevisiae*	
Astaxanthin	Overexpress native *idi* and *gps* from *Archaeoglobus fulgidus* and express the gene cluster *crtBIYZW* from *Agrobacterium aurantiacum*	1.4 mg/g dcw (50-fold) (Wang et al., 1999)	*E. coli*	*Haematococcus* microalgae 1.5–3.0% by dry weight (Lorenz and Cysewski, 2000)	Mixture of isomers, not approved for human consumption (Li et al., 2011)
	Overexpress *idi* and *dxs* and balance expression of *crtE*, *crtB*, *crtI*, *crtY*, and *crtZ* from *Pantoea ananatis* and *crtW148* (NpF4798) from *Nostoc punctiforme*, which were inserted into the chromosome	1.4 mg/g dcw (20-fold) (Lemuth et al., 2011)	*E. coli*	
Levopimaradiene	Combinatorially mutate the GGPPS–LPS pathway	700 mg/l (2,600-fold) (Leonard et al., 2010)	*E. coli*	Young *Ginkgo biloba* trees 1–7 mg/g dry weight (Matsuda and Schepmann, 2008)	<3% Overall yield (Matsuda and Schepmann, 2008)
Lycopene^2^	Express *Erwinia* carotenoid biosynthesis gene cluster and *idi* from *Haematococcus pluvialis*	1.03 mg/g dcw (4.5-fold) (Kajiwara et al., 1997)	*E. coli*	Tomatoes 0.15–0.25 mg/g (Rath, 2009)	0.13 mg/g and 70% trans configurations (Olempska-Beer, 2006)
	Redesign the global regulatory system, the Ntr regulon	160 mg/l (from 0 mg/l) (Farmer and Liao, 2000)	*E. coli*	94–96% trans configurations (Olempska-Beer, 2006)	
	Overexpress the catalytic domain of HMG and disrupt *ERG9*	7.8 mg/g dcw (7-fold) (Shimada et al., 1998)	*C. utilis*	
	Overexpress genes identified by the FSEOF strategy combined with gene knockouts	12.32 mg/g dcw (4-fold) (Choi et al., 2010)	*E. coli*	
	Overexpress and knockout genes selected from a metabolic landscape	16 mg/g dcw (4-fold) (Jin and Stephanopoulos, 2007)	*E. coli*	
	Use “global transcription machinery engineering” to improve phenotypes	7.7 mg/l (1.8-fold) (Alper and Stephanopoulos, 2007)	*E. coli*	
	Optimize DXP pathway with “multiplex automated genome engineering”	9 mg/g dcw (5-fold) (Wang et al., 2009)	*E. coli*	
Miltiradiene	Fuse SmCPS and SmKSL as well as BTS1 and ERG20	365 mg/l (340-fold) (Zhou et al., 2012)	*S. cerevisiae*	*Salvia miltiorrhiza <*40 mg/g dry weight (Li et al., 2012)	4 mg/ml of the precursor salvianolic acid B (Gu et al., 2008)
Patchoulol	Replace the native *ERG9* promoter with the methionine repressible *MET3* promoter	16.9 mg/l (1.5-fold) (Asadollahi et al., 2008)	*S. cerevisiae*	*Pogostemon cablin* 30–40% total mass (Hybertson, 2007)	6% Overall yield of the precursor norpatchoulenol (Kolek et al., 2009)
	Fuse the native farnesyl diphosphate synthase and the heterologous patchoulol synthase and repress *ERG9*	40.9 mg/l (2-fold) (Albertsen et al., 2011)	*S. cerevisiae*	
Taxadiene	Express genes for GGPPS, taxadiene synthase, three cytochrome P450 hydroxylases, and three acyl/aroyl CoA dependent transferases and build a five step taxoid pathway	1 mg/l (100-fold) (Dejong et al., 2006)	*S. cerevisiae*	*Taxus brevifolia* 0.01–0.1% dry bark weight (Hezari et al., 1995)	18–20% Overall yield (Mendoza et al., 2012)
	Express genes for geranylgeranyl diphosphate synthase from *Sulfolobus acidocaldarius* and a codon-optimized taxadiene synthase from *Taxus chinensis*	8.7 mg/l (40-fold) (Engels et al., 2008)	*S. cerevisiae*	
	Vary small pathway modules simultaneously to determine the optimally balanced complete pathway (“multivariate modular pathway engineering”)	1 g/l (15,000-fold) (Ajikumar et al., 2010)	*E. coli*	
Zeaxanthin^2^	Overexpress different combinations of *idi* from *Xanthophyllomyces dendrorhous*, *dxr* from *Sulfolobus acidocaldarius*, and native *dxs*	1.6 mg/g dcw (3.5-fold) (Albrecht et al., 1999)	*E. coli*	*Tagetes erecta’s* red flowers 23% dry weight (Stankovic, 2004)	12% Overall yield of racemic mix (Khachik and Chang, 2009)
	Use the “ordered gene assembly in *Bacillus subtilis* (OGAB) method” to determine optimal gene order	820 μg/g dcw (4.4-fold) (Nishizaki et al., 2007)	*E. coli*	
α-Santalene	Replace the native *ERG9* promoter with the glucose-responsive *HXT1* promoter, delete the genes for lipid phosphate phosphatase and pyrophosphate phosphatase, and overexpress a truncated 3-hydroxyl-3-methyl-glutaryl-CoA reductase	0.21 mg/g dcw (3.4-fold) (Scalcinati et al., 2012)	*S. cerevisiae*	*Santalum album* 1–2% by weight of oil (Jones et al., 2011)	8% Overall yield (Bastiaansen et al., 1996)
β-Carotene^2^	Overexpress different combinations of *idi* from *Xanthophyllomyces dendrorhous*, *dxr* from *Sulfolobus acidocaldarius*, and native *dxs*	1.5 mg/g dcw (3.5-fold) (Albrecht et al., 1999)	*E. coli*	Mostly *Dunaliella salina* 300 mg/m^2^/day (Hosseini Tafreshi and Shariati, 2009)	85% Yield using triphenyl-phosphine oxide, which is harmful to aquatic organisms (USDA, 2011)
	Replace the native promoters for the chromosomal genes *dxs*, *ispDispF*, *idi*, and *ispB* with strong T5 bacteriophage promoters	6 mg/g dcw (24.5-fold) Yuan et al., 2006)	*E. coli*	

*Dcw, dry cell weight*.

*^1^Acronyms are defined in the main text*.

*^2^Currently produced or produced in the near term by microbial biosynthesis*.

## Isoprenoid Pathway

**Figure 1:**
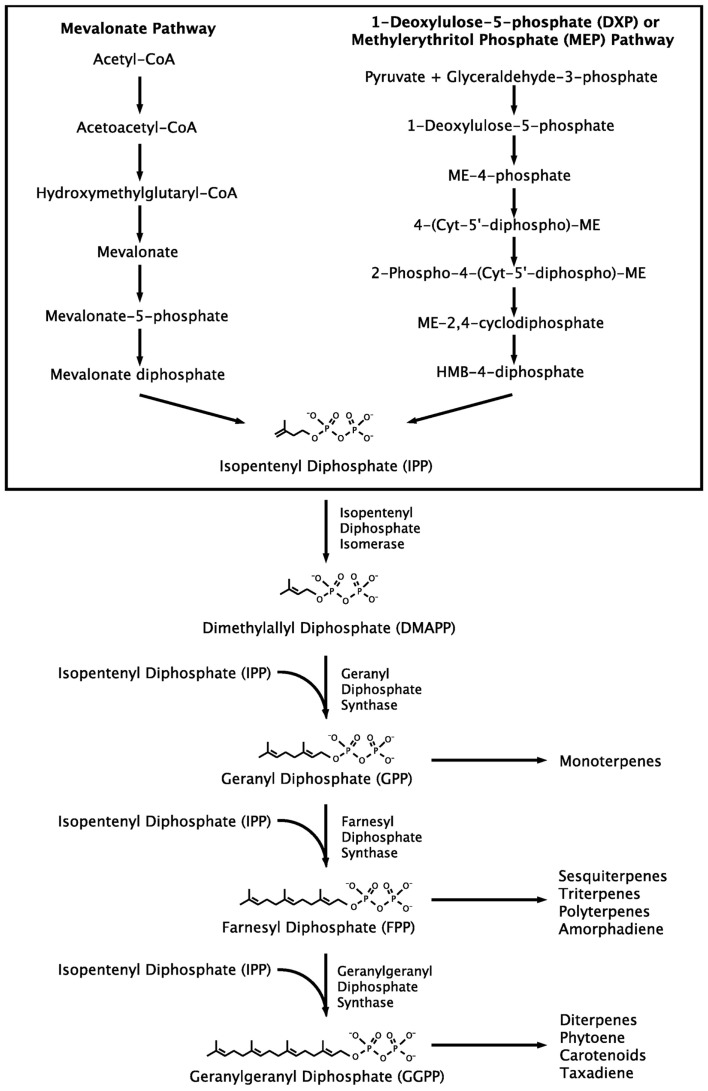
**Isoprenoid pathway**. Two distinct pathways for IPP production are shown together, but they exist in different organisms.

Although isoprenoids include a wide range of compounds, they are synthesized through a common metabolic pathway. The isoprenoid pathway (Figure 1) begins with the conversion of acetyl-CoA to isopentenyl diphosphate (IPP). IPP is then isomerized to dimethylallyl diphosphate (DMAPP), which forms geranyl diphosphate (GPP), then farnesyl diphosphate (FPP), followed by geranylgeranyl diphosphate (GGPP). At this point different isoprenoids begin to branch off into individualized pathways (Kajiwara et al., 1997; Schmidt-Dannert, 2000; Walter and Strack, 2011). Two distinct pathways exist for the production of the precursor compounds IPP and DMAPP, the mevalonate pathway, and the methylerythritol phosphate (MEP) pathway. Thus, researchers enjoy multiple options when selecting the metabolic pathway for production of the chosen isoprenoid (Chang and Keasling, 2006). Furthermore, the isoprenoid pathway has been expressed in a variety of hosts and assembled using genes from a diversity of sources (Misawa and Shimada, 1997; Schmidt-Dannert, 2000; Das et al., 2007; Nishizaki et al., 2007; Maury et al., 2008).

The IPP precursor supply has been engineered through several techniques to improve the commercial viability of isoprenoid biosynthesis. Martin et al. (2003) began their work on the synthesis of the sesquiterpene artemisinin by assembling *S. cerevisiae*’s mevalonate pathway into two operons that were co-expressed in *E. coli*. Post-transcriptional processes made balanced expression of genes within operons difficult. To overcome this problem, libraries of “tunable intergenic regions” (TIGRs) and recombined control elements (RBS sequestering sequences, mRNA secondary structures, and RNase cleavage sites) were screened to select the *E. coli* strain that produced sevenfold more mevalonate (Pfleger et al., 2006). Shiba et al. amplified the precursor flux to the mevalonate pathway in *S. cerevisiae* by overexpressing acetaldehyde dehydrogenase and incorporating *Salmonella enterica’s* acetyl-CoA synthetase. This kept more carbon flow in the cytosol, as opposed to the mitochondria, resulting in more mevalonate (Shiba et al., 2007). To increase lycopene production by boosting the precursor supply, a synthetic mevalonate pathway was assembled in *E. coli*, which included yeast mevalonate kinase (yMVK), human 5-phosphomevalonate kinase (hPMK), yeast 5-diphosphomevalonate decarboxylase (yPMD), and *E. coli* IPP/DMAPP isomerase (Rodriguez-Villalon et al., 2008). Dueber et al. (2009) created synthetic protein scaffolds to co-localize mevalonate pathway enzymes, leading to a 77-fold improvement in product titers. This approach demonstrated that high production of mevalonate can be achieved with low enzyme expression and reduced metabolic burden.

## Carotenoids

Carotenoids are among the first natural products whose titers were improved through synthetic biology tools. Early work on the microbial production focused on increasing the supply of intermediates in the first steps of the isoprenoid pathway. In 1997, the production of carotenoids in *E. coli* was improved by a factor of 2.7, for a total of 1.3 mg/g dry cell weight (dcw), by introducing heterologous genes for IPP isomerase (Kajiwara et al., 1997). Albrecht et al. (1999) increased the nutrients β-carotene and zeaxanthin 3.5-fold, to reach 1.5 and 1.6 mg/g dcw respectively, by overexpressing different combinations of the genes for IPP isomerase from *Xanthophyllomyces dendrorhous*, GGPP synthase from *Sulfolobus acidocaldarius*, and native DXP synthase. More than a 50-fold improvement was made in the production of astaxanthin, used to treat several degenerative nerve diseases, by boosting IPP and GGPP formation as well as expressing the gene cluster *crtBIYZW* from *Agrobacterium aurantiacum* in *E. coli* for a total of 1.4 mg/g dcw (Wang et al., 1999). However, unhindered metabolite production can lead to metabolic imbalance. Farmer and Liao (2000) redesigned a global regulatory system in *E. coli* to allow lycopene production only in the presence of sufficient glucose, as indicated by acetyl phosphate availability. This control loop decreased the metabolic imbalance, thus increasing the final yield of the nutritional supplement. Shimada et al. (1998) also improved lycopene production by focusing on the later steps of the isoprenoid pathway. In *Candida utilis* that expressed exogenous *crtE*, *crtB*, and *crtI*, the gene*ERG9*, which diverts FPP to the ergosterol pathway, was disrupted and the catalytic domain of HMG was overexpressed, resulting in 7.8 mg lycopene/g dcw. Novel carotenoids were created in *E. coli* through the expression of mutagenic libraries of phytoene desaturase and lycopene cyclase, enzymes that regulate branchpoints in the later stages of carotenoid biosynthesis. A wide variety of metabolites, including 3,4,3′,4′-tetradehydrolycopene, a fully conjugated carotenoid, and torulene, a new cyclic carotenoid, were observed (Schmidt-Dannert et al., 2000).

More recently, powerful new techniques have been employed to further improve carotenoid production. Jin and Stephanopoulos (2007) constructed a lycopene production metabolic landscape using *E. coli* strains that incorporated different combinations of overexpression and knockout targets. From this landscape, the best combination of genes increased lycopene production to 16 mg/g dcw. β-carotene production reached 6 mg/g dcw by replacing the native *E. coli* promoters for the chromosomal genes *dxs*, *ispDispF*, *idi*, and *ispB* with strong T5 bacteriophage promoters. Modifying chromosomal genes, instead of introducing high-copy vectors to overexpress the target genes, decreased the metabolic burden (Yuan et al., 2006). Lemuth et al. also used similar techniques by balancing expression of *crtE*, *crtB*, *crtI*, *crtY*, and *crtZ* from *Pantoea ananatis* and *crtW148* (NpF4798) from *Nostoc punctiforme*, which were inserted into the chromosome of *E. coli*. This plasmid-free strain created astaxanthin as its only carotenoid at 1.4 mg/g dcw (Lemuth et al., 2011). Using the “ordered gene assembly in *Bacillus subtilis* (OGAB) method” to put together multiple genes in a single step, Nishizaki et al. (2007) determined the optimum gene order which subsequently produced 820 μg zeaxanthin/g dcw. The “flux scanning based on enforced objective flux” (FSEOF) strategy identified targets for gene amplification that were not intuitive. When combined with gene knockouts, 12.32 mg lycopene/g dcw was achieved (Choi et al., 2010). Alper and Stephanopoulos (2007) randomly mutated the *E. coli* sigma factor σ^70^ to look for desired complex phenotypes. This “global transcription machinery engineering” (gTME) improved lycopene production. “Multiplex automated genome engineering” (MAGE) was proposed by Wang et al. They modified 24 genetic components at once from a degenerate pool of synthetic DNA, achieving a fivefold increase in lycopene production in just 3 days (Wang et al., 2009).

## Artemisinin

The microbial production of the potent anti-malaria drug artemisinin has utilized a number of advances in the synthetic biology field. Balancing metabolic flux with the codon-optimized amorphadiene synthase gene improved the titer of amorphadiene, an artemisinin precursor, beyond what had been accomplished by increasing IPP precursor supply (Martin et al., 2003). Ro et al. (2006) redesigned the mevalonate pathway in *S. cerevisiae* to increase production of FPP and introduced *Artemisia annua’s* amorphadiene synthase and cytochrome P450 for the final oxidation steps. The modifications resulted in 100 mg/l of artemisinic acid (Ro et al., 2006). Anthony et al. (2009) achieved the amorphadiene titer of 293 mg/l by identifying the limiting reaction enzymes and balancing gene expression through plasmid copy number and promoter strength. Building upon all of the previous works in the Keasling lab, production of >40 g/l amorphadiene was achieved by overexpressing every enzyme in the mevalonate pathway and modifying fermentation conditions. Subsequently, the amorphadiene was chemically converted to dihydroartemisinic acid, the precursor of the antimalarial agent artemisinin (Westfall et al., 2012). *A. annua’s* amorphadiene synthase, codon-optimized and placed under the control of the *rpsJ* promoter, and the native FPP synthase were expressed in a genome-minimized strain of *Streptomyces avermitilis*. This approach led to heterologous biosynthesis of 30 mg/l of amorphadiene while not producing any of the major endogenous secondary metabolites (Komatsu et al., 2010). Farhi et al. (2011) co-localized heterologous FDP synthase and amorphadiene synthase to the mitochondria to improve the amorphadiene titer by 20-fold, for a total of 20 mg/l.

## Diterpenes and Other Sesquiterpenes

Biosynthetic pathways for various diterpenes and sesquiterpenes have also been engineered for improved production through synthetic biology. To maximize production of several sesquiterpenes, Asadollahi et al. replaced the native*ERG9* promoter, which is responsible for diverting the terpenoid precursor FPP to a competing pathway, with the methionine repressible *MET3* promoter. After optimizing methionine levels, 16.9 mg/l of patchoulol, the starting compound in the chemical synthesis of the chemotherapeutic drug paclitaxel (Taxol), was achieved (Asadollahi et al., 2008). Scalcinati et al. chose to control *ERG9* expression by coupling it with the glucose-responsive *HXT1* promoter. In addition to using this promoter, the genes encoding lipid phosphate phosphatase and pyrophosphate phosphatase were deleted, and a truncated 3-hydroxyl-3-methyl-glutaryl-CoA reductase (HMGR) was overexpressed to produce α-santalene, a skin cancer chemopreventative, at 0.21 mg/g dcw (Scalcinati et al., 2012). The native FPP synthase and the heterologous patchoulol synthase were fused to reduce metabolic diffusion distance between enzymes, increasing patchoulol production twofold, to a total of 40.9 mg/l, in *S. cerevisiae* (Albertsen et al., 2011). Miltiradiene, related to the Chinese medicinal herb *Salvia miltiorrhiza*, was produced up to 365 mg/l in a 15 l bioreactor, by fusing labdadienyl/copalyl diphosphate synthase (SmCPS) and kaurene synthase-like (SmKSL) as well as GGPP synthase (BTS1) and FPP synthase (ERG20) in *S. cerevisiae* (Zhou et al., 2012). The capacity of downstream pathways can also limit titers. The geranylgeranyl diphosphate synthase – levopimaradiene synthase (GGPPS – LPS) pathway was combinatorially mutated to accommodate the engineered upsurge in precursors. This approach led to a 2,600-fold increase, for a total of 700 mg/l, of the diterpene levopimaradiene, used to produce the ancient medicinal ginkgolides (Leonard et al., 2010).

## Paclitaxel

Application of synthetic biology tools to microbial production of the cancer chemotherapy drug paclitaxel will decrease its cost and increase its availability. Paclitaxel, known as Taxol, is a potent chemotherapy drug, which is very difficult to chemically synthesize (Chandran et al., 2011) and is extracted at very low efficiency from the bark of the rare Pacific yew (Ajikumar et al., 2008). Dejong et al. (2006) were the first to express genes for a portion of the Taxol pathway in *S. cerevisiae*, but production levels of the Taxol intermediate, taxadiene, were low. Several changes to taxadiene synthesis in yeast were introduced, including an alternate geranylgeranyl diphosphate synthase from *S. acidocaldarius* and a codon-optimized taxadiene synthase from *Taxus chinensis*, ultimately resulting in a 40-fold titer increase to 8.7 mg/l (Engels et al., 2008). Using *E. coli* as a host, Ajikumar et al. (2010) divided the metabolic pathway into smaller modules and varied the expression levels simultaneously to determine the optimally balanced pathway without requiring high throughput screening. This “multivariate modular pathway engineering” resulted in the taxadiene titer of 1 g/l. Although challenges remain for the biosynthesis of Taxol and other compounds, the range of advancements in isoprenoid production by microbial biosynthesis shows promise for increasing their availability at reduced cost.

## Conclusion

The past decade has witnessed the potential of synthetic biology to make the microbial isoprenoid production become industrially relevant. However, further improvements in yield and expansion to new medically important compounds can be attained through the development of additional tools. An incomplete understanding of the complexity of biosynthetic pathways limits the ability to fully forward engineer microbial production (Nielsen and Keasling, 2011; Stephanopoulos, 2012). Continued innovations in systems biology to elucidate the complex regulatory and metabolic networks will advance the predictive potential of mathematical models, and therefore the ability to generate optimized microbial cell factories (Jarboe et al., 2010; Nielsen and Keasling, 2011; Keasling, 2012). Genome mining, scanning genome sequences for natural functions, will accelerate the rate of new compound discoveries. Improved enzyme engineering will also support the *de novo* design of biosynthetic pathways (Ajikumar et al., 2008; Jarboe et al., 2010). Moreover, biological devices built from well characterized and standardized genetic parts can be used to control metabolic pathways. Incorporation of these strategies would lead to engineered microbes for industrial-scale production of medically important compounds.

## Conflict of Interest Statement

The authors declare that the research was conducted in the absence of any commercial or financial relationships that could be construed as a potential conflict of interest.
